# Development of double-layer FF peptide microrod arrays for high performance piezoelectric nanogenerators

**DOI:** 10.1016/j.fmre.2024.07.009

**Published:** 2024-08-05

**Authors:** Jiaojiao Zhang, Jing Liu, Wen Hu, Xue Jiang, Long Zhou, Yumin Tang, Zhong Lin Wang, Rusen Yang

**Affiliations:** a School of Advanced Materials and Nanotechnology, Xidian University, Xi'an 710071, China; b Zhejiang Cachi New Energy Technology Co., Ltd., Huzhou 313100, China; c Beijing Institute of Nanoenergy and Nanosystems, Chinese Academy of Sciences, Beijing 101400, China; d School of Materials Science and Engineering, Georgia Institute of Technology, Atlanta, GA 30332-0245, United States

**Keywords:** Diphenylalanine (FF), Peptide, Piezoelectric nanogenerator, Polarization, Microrod

## Abstract

Piezoelectric biomaterials have shown good energy conversion capability and promising future for biomedical applications. However, their performance is still limited by their relatively low piezoelectric constant, and increasing the power by connecting multiple devices is restricted by the challenge of synchronizing all individual devices. Herein, we develop double-layer FF peptide microrods arrays with independently controlled polarization in each layer. The resultant piezoelectric nanogenerator showed much enhanced performance because the synchronous deformation and the appropriate polarization directions of microrods in each individual layer enable the constructive contribution of voltage and current output from all microrods. The nanogenerator generated an open circuit voltage of 2.05 V in a serial connection mode, which doubles the output from a single-layer device. When two layers are connected in parallel and the polarization is in a head-to-head configuration, a twofold increase in the current output is also achieved. This work provides a new strategy to design integrated devices with much improved performance for wearable technology and therapeutic systems.

## Graphical abstract

The double-layer FF peptide microrods arrays with independently controlled polarization in each layer were developed. The resultant piezoelectric nanogenerator showed much enhanced performance because the synchronous deformation and the appropriate polarization directions of microrods in each individual layer enable the constructive contribution of voltage and current output from all microrods.

**Figure:**
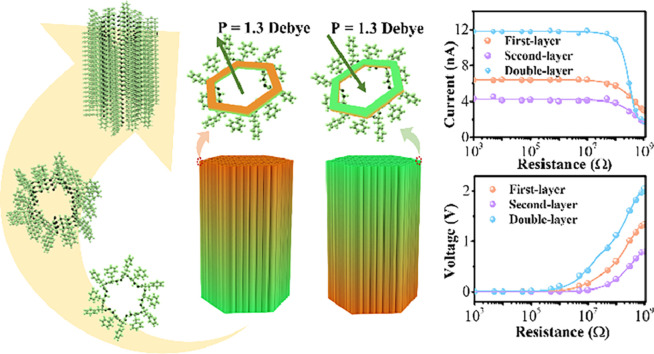

## 1 Introduction

Piezoelectric biomaterials with exceptional biodegradability, accessibility, and biocompatibility have gained increasing attention because of their potential applications in energy harvesting, implantable sensors, and neural tissue engineering [[1], [2], [3], [4], [5], [6], [7], [8]]. Notably, numerous natural biomaterials, such as hair [6], bones [9], collagens [10], viruses [11], celluloses [12], tendons [13], and skin [14], have been found to exhibit piezoelectric properties. However, their weak piezoelectric coefficients limit their potential biomedical applications. To overcome these issues, researchers focus on short peptides that can spontaneously self-assemble into highly-ordered superstructures with specific functionalities by non-covalent interactions [[15], [16], [17], [18], [19]]. For instance, diphenylalanine (FF) is assembled into nanotubes, nanowires, and nanospheres by solution and physical vapor deposition methods. The FF nanotubes with a space group P6_1_ (noncentrosymmetric) show a shear piezoelectric constant d_15_ of 60 pm V^−1^, which has great potential for energy harvesting applications [20]. However, the nanostructures with different polarization directions limit their application.

If the polarization direction of the nanostructure is unified during self-assembly, the output performance of the piezoelectric nanogenerator (PENG) is improved [21,22]. Lee et al. used the meniscus-driven self-assembly process to obtain aligned FF nanotubes, achieving high output performance of FF nanotube-based PENG [22]. Motai et al. reported a technique for forming thin films of crystallized amino acids and dipeptides by solution shearing [23]. Yuan et al. developed ordered valine array structures via physical vapor deposition, and the PENG based on the valine array is 4.6 times higher than that of nanogenerators based on powder valine crystals [24]. Despite developing various self-assembly techniques for piezoelectric biomaterial synthesis, most of these methods present challenges in achieving the highly aligned structures with uniform polar orientation. Our previous research has demonstrated that the FF peptides self-assemble into uniformly polarized microrod arrays when an electric field is applied during self-assembly. The mobile FF molecules in the solution are aligned so that their electrical dipoles are along the applied electric field during the FF peptide self-assembly. After the growth process is completed, the polarization remains stable and does not switch by a high electric field [25]. Furthermore, FF peptide microrod arrays achieve an enhanced piezoelectric constant d_33_ with the value of 17.9 pm V^−1^. The FF peptides are increasingly recognized as promising materials for building PENGs that can harvest environmental energy. However, the power output of PENG is limited by the piezoelectric potential across the length of one microrod and the total charges contributed from all microrods. Therefore, new strategies are needed to enhance the voltage and the current output of the device for its further development.

Herein, we demonstrate a facile way to successfully fabricate double-layer structured FF peptide microrods through the epitaxial growth method and design an integrated and double-layer structured PENG based on aligned FF peptide microrods to enhance the output performance. Two layers of FF microrod arrays can be achieved with one layer on top the other. Each layer has uniform polarization, and the polarization direction in each layer can be independently controlled. PENGs can be fabricated with much enhanced voltage output or current output, depending on the relationship between the polarization direction of these two FF microrod arrays.

## 2 Results and discussion

**Fig. 1:**
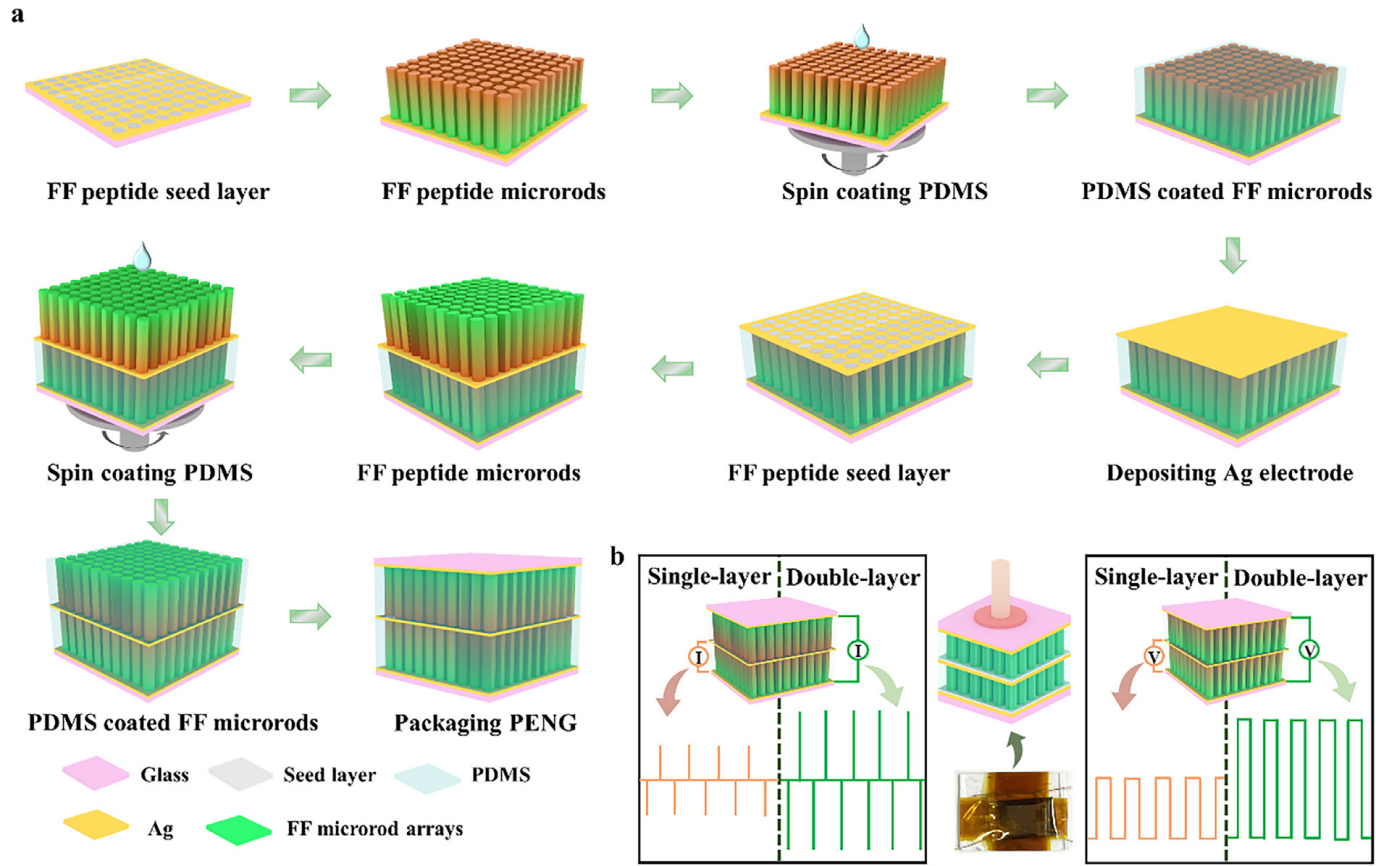
**Preparation of double-layer structured FF peptide microrods for energy harvesting**. (a) Schematic illustration of the fabrication of a PENG based on double-layer structured FF peptide microrods. (b) Twofold increase in voltage or current outputs of the double-layer structured nanogenerators.

The preparation process for a PENG based on the double-layer structured FF peptide microrods is shown in Fig. 1a. The double-layer structured FF peptide microrods are prepared through the epitaxial growth method [[25], [26], [27]]. Briefly, a seed layer is formed on a substrate in the circulation chamber. FF molecules self-assemble on the seed layer in FF aqueous solution to form microrod arrays that are oriented vertically to the substrate. An electric field parallel or antiparallel to the normal direction of the substrate surface is applied during the self-assembly process (Fig. S1). The double-layer structured FF peptide microrods are obtained following the two-step epitaxial growth process. The polydimethylsiloxane (PDMS) insulating layer has a twofold role, serving both as a protective layer to absorb mechanical impact and as a support layer for the growth of the second layer of microrods. The polarization of FF microrods in each layer can be individually controlled, as the polarization of the first layer won't be influenced by the electric field applied for the second layer growth [25]. The obtained PENG is utilized in living environments, such as harvesting mechanical energy (Fig. 1b). The output performance of the double-layer structured FF peptides is superior to that of the single-layer structured FF peptides.

**Fig. 2:**
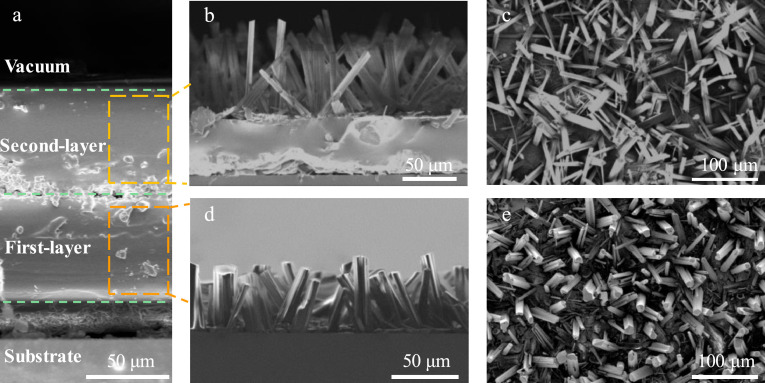
**Characterization of the FF seed layer and FF peptide microrods**. (a) SEM image of the cross-section of the double-layer structured FF peptide microrods. (b) Cross-section image of the second layer of FF peptide microrods. (c) Top view SEM image of the second layer of FF peptide microrods. (d) Cross-section image of the first layer of FF peptide microrods. (e) Top view SEM image of the first layer of FF peptide microrods.

A cross-sectional SEM image of the double-layer structured FF peptide microrods coated with PDMS is shown in Fig. 2a, revealing that the thickness of the double-layer structured FF peptide microrods is approximately 170 µm. The FF peptide microrods can be visualized as a tightly packed cluster of hydrophobic nanotubes with a hydrophilic hollow channel. During the self-assembly process, six FF molecules form ring structures around the water molecule through hydrogen bonds between the FF molecules. These rings self-assemble into tubes by stacking the aromatic side chains and hydrogen bonding of the peptide backbones [[28], [29], [30], [31], [32]]. A highly textured and crystallized seed layer with a preferred orientation perpendicular to the substrate is indispensable for achieving epitaxial growth of FF peptide microrods with the desired alignment.

The content of water molecules is crucial in the self-assembly process of FF molecules. The seed layers crystallized under different relative humidities are analyzed by micromorphology. As shown in Fig. S2a-c, FF molecules are crystallized when the relative humidity is > 90%. Regular hexagonal crystals with dense and vertical crystallinity can be formed by growing the FF seed layer at a relative humidity of approximately 100%. In addition, the temperature of the circulating room is critical to the activity of the FF molecules and the evaporation rate of the HFIP solvent. When the temperature of the circulating room surpasses 30 °C, the movement of the FF molecules increases, and the HFIP solvent evaporates quickly, resulting in supersaturation and rapid crystallization of FF molecules. It is difficult for water molecules to diffuse downward, so the FF molecules tend to crystallize horizontally (Fig. S2d-f). Therefore, the first-layer structured seed layer exhibits good vertical crystallization when the relative humidity in the circulating chamber is 100% and the temperature is at 30 °C. This highly vertical crystalline seed layer proves advantageous for the epitaxial growth of FF peptide microrods. The FF peptide microrods grown from the first-layer structured microrods are presented in Fig. 2d,e, indicating the formation of the vertically ordered FF peptide microrods. The diameter of the FF microrods ranges from about 2 to 13 µm, and the length is approximately 50 µm.

A flat intermediate electrode and precise relative humidity and temperature control are required to fabricate the second FF seed layer with high crystallinity. As shown in Fig. S3, after spinning PDMS on the surface of the first-layer structured FF peptide microrods and depositing an Ag electrode, a smooth surface is obtained, which is the basis for the subsequent growth of the second FF seed layer. The second FF seed layer is obtained at a temperature of 30 °C with 100% humidity (Fig. S4). The second layer of FF peptide microrods is grown from the second FF seed layer and exhibits a vertically arranged structure with a diameter ranging from 2 to 12 µm, and a length is approximately 65 µm (Fig. 2b,c). The vertical alignment, density, and height difference is observed between the FF peptide microrods in the first and second layers. This difference may be caused by the alterations and fluctuation in experimental conditions, such as the seed layers and minor discrepancies in experimental parameters, such as temperature and humidity.

**Fig. 3:**
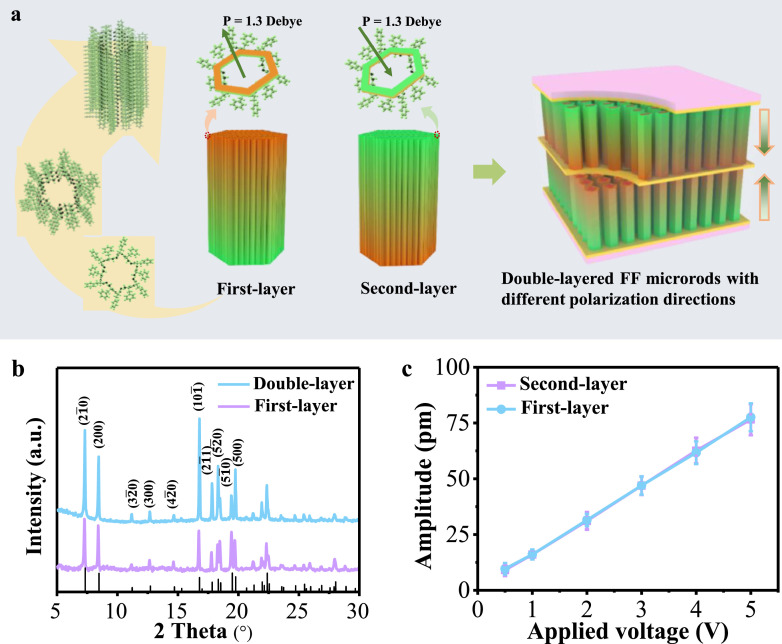
**Characterization of double-layer structured FF microrods with opposite electric fields applied for the first and the second layers**. (a) Schematic diagrams of the self-assembly process of the double-layer structured microrods. (b) XRD of the first layer and the double layer of FF peptide microrods. (c) Linear dependence of the PFM amplitude on the applied voltage for FF microrods from the first layer and from the second layer.

The self-assembly process of the FF molecules by applying an electric field is shown in Fig. 3a. Six FF molecules form an FF ring, which translates axially to form a tube. The FF peptide microrods are considered as a stack of these nanotubes. The FF ring has a positive electricity center composed of six amino groups and a negative electricity center composed of six carboxyl groups. Each FF ring exhibits a dipole moment P_s_ of ∼1.3 Debye perpendicular to the ring plane [33]. The newly incorporated FF rings typically show a matching orientation to the pre-existing FF rings in the crystal for energy minimization. When an electric field is applied, it plays a critical role in driving the adjustment of FF molecule orientations in the self-assembly process [21]. The FF microrods exhibit either upward or downward polarization under positive or negative electric field growth. After the growth process is finished, the polarization remains highly stable and resistant to change, even in the presence of a strong electric field [25]. Therefore, when the first layer and the second layer of FF peptide microrods are grown with a positive and negative electric field during growth, they exhibit opposite polarization directions. Hence, the multiple layer structured FF peptide microrods can be achieved with the polarization of each layer controlled independently, which significantly facilitates the design of FF-based devices with enhanced performance.

The crystal structures of both the single-layer FF microrods and the double-layer FF peptide microrods are characterized by XRD analysis, revealing a hexagonal structure with a space group P6_1_ (Fig. 3b). Strong diffraction peaks corresponding to the (10$\bar{1}$), (2$\bar{1}$0), (200), and other planes are detected, indicating high crystallinity. AFM measurements on the FF peptide microrods show a linear relationship between the amplitude and the applied voltage (Fig. 3c). The slope gives an effective piezoelectric coefficient d_33_ of 15 pm V^−1^ for both the single-layer microrod and the double-layer structured FF peptide microrod. In addition, the piezoelectric constant d_33_ is measured using a quasi–static piezoelectric d_33_–meter. As shown in Fig. S5, the piezoelectric constant d_33_ of FF microrods from the first layer and from the second layer are 14.1 pC/N and 14 pC/N, respectively. The results are consistent with the AFM test results. These results demonstrate that the double-layer structured FF peptide microrods have the potential for mechanical energy conversion applications.

**Fig. 4:**
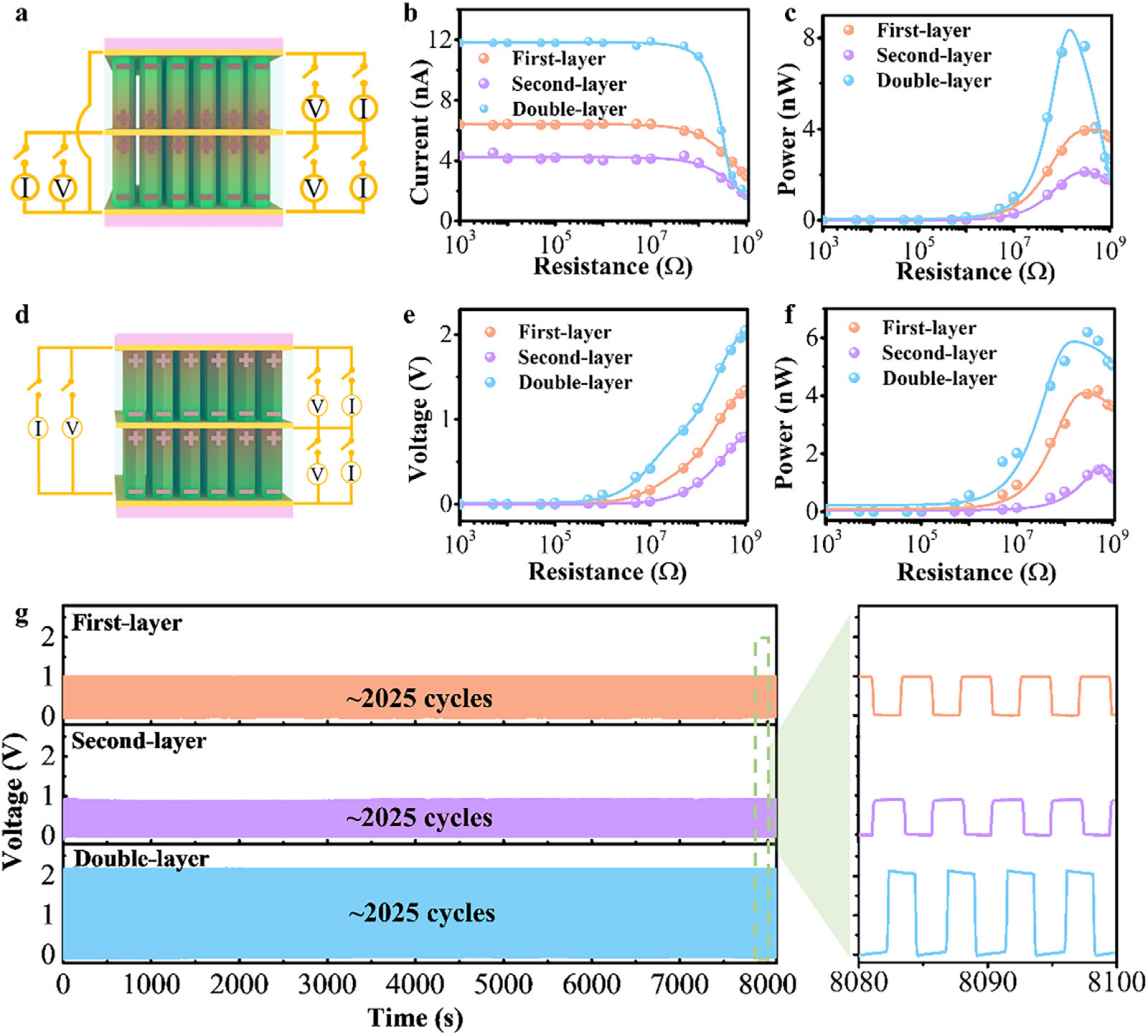
**The output performance of double-layer structured PENG devices**. (a) Schematic diagrams of the double-layer structured PENG based on FF microrods with opposite polarization directions (head-to-tail) in adjacent layers. (b) Output currents and (c) powers of the first layer, the second layer, and the double layer connected to different load resistances. (d) Schematic diagrams of the double-layer structured PENG based on FF microrods with the same polarization direction (head-to-head) in adjacent layers. (e) Output voltages and (f) powers of the first layer, the second layer, and the double layer connected to different load resistances. (g) Stability of the double-layer structured PENGs shown in (d).

To explore the interaction of the electromechanical coupling of two layers of microrod arrays and their application potential, we examined the performance of PENGs based on the double-layer structured FF microrods. As shown in Fig. 4a, the double-layer structured FF peptide microrods were achieved with the first layer grown with a positive electric field and the second layer with a negative electric field and sandwiched between three electrodes connected in parallel to an external load or a test instrument. The first-layer, the second-layer, and the double-layer of FF peptide microrods generate open-circuit voltages of 1.05 V, 1 V, and 1.25 V, respectively under a cyclic compression force of 28 N (Fig. S6). The short-circuit current for these three cases are 6.82 nA, 4.79 nA, and 12.6 nA (Fig. S6). Notably, the output current obtained from the PENG based on the double-layer structured FF peptide microrods is approximately equal to the sum of the PENGs based on the outputs of the first-layer and second-layer structured FF peptide microrods. This indicates that all microrods in both layers are synchronized in motion and contribute constructively to the current output of the PENG devices, while the output voltage is still determined by the piezoelectric potential across the length of one microrod. The reverse-connected PENGs show reversed voltages, suggesting that the measured output signals are truly produced by the PENGs (Fig. S7).

The output voltages and currents exhibit a linear increase in response to mechanical forces, indicating a linear piezoelectric response (Fig. S8). When the PENGs are connected to external resistors, the output voltages and currents vary with different load resistances under a cyclic compression force of 28 N, as shown in Fig. 4b and Fig. S9. As the resistances increase, the output voltages increase and the output currents decrease. The output powers of the PENGs are given in Fig. 4c, indicating that the maximum instantaneous power of the PENG based on the double-layer structured FF peptide microrods reaches up to 7.63 nW at the 300 MΩ load resistance, which is 3.6 times higher than the maximum instantaneous power of the PENG based on one layer FF peptide microrods. The stability of the output is tested, and the results show a stable current output for > 2025 cycles (Fig. S10).

Furthermore, the double-layer structured FF peptide microrods with a positive electric field applied to all layer growth are connected to an external load or a test instrument, as shown in Fig. 4d. Performance of PENGs based on the first-layer, the second-layer, and the double-layer structured FF peptide microrods are carried out under a cyclic compression force of 28 N. The open-circuit voltages from the first layer and the second layer are 0.98 V and 0.91 V, respectively. However, the PENG based on the double-layer structured FF peptide microrods exhibits an open-circuit voltage of 2.05 V (Fig. S11), which is approximately the sum of the voltages produced from individual layers. This indicates that PENGs based on the double-layer structured FF peptide microrods with the same polarization direction can enhance their voltage output through serial connection modes. The short-circuit currents of PENGs based on the first-layer, second-layer, and double-layer structured FF peptide microrods are approximately similar, at 4.23 nA, 4.31 nA, and 4.37 nA, respectively (Fig. S11). Switching-polarity tests are performed to verify that the measured output signals [24,34], and negative output voltages are obtained when the PENGs are reversed (Fig. S12). The output voltages and currents exhibit a linear increase in response to mechanical forces, indicating a linear piezoelectric response (Fig. S13).

It is critical to assess the instantaneous powers of PENGs based on the FF peptide microrods, which are measured through various load resistances ranging from 1000 Ω to 1 GΩ. As the resistances increase, the output voltages increase and the output currents decrease (Figs. 4e and S14). The maximum instantaneous power of PENGs based on the double-layer structured FF peptide microrods reaches up to 6.2 nW at the 300 MΩ load resistance, which is higher than the power output only from the first layer or from the second layer (Fig. 4f). Stability is essential for practical applications. As shown in Fig. 4g, there is no significant reduction in the output voltage for over 2025 cycles, indicating good stability of the FF peptide microrods. These results demonstrate that output voltages can be enhanced by integrating PENGs in serial connections and output currents can be enhanced by integrating PENGs in parallel connections.

Each microrod within the compressed region operates as a charging pump, resulting in the efficient accumulation of output voltages or currents when the pressing and releasing processes of multiple microrods are harmoniously synchronized during mechanical deformation [35]. As shown in Figs. S5,S10, the first-layer, second-layer, and double-layer of FF peptide microrods have similar output voltage and circuit waveforms with the same frequency and phases. Additionally, the double-layer structured FF microrods are 1–3 type piezoelectric composites, which improve the mechanical performance and electromechanical coupling characteristics of devices [36]. The quantity of microrods is critical in generating piezoelectric potential, which enhances energy-harvesting performance via a double-layered design. Therefore, the piezoelectric energy-harvesting properties could be improved by adopting this double-layer structure, resulting in an almost two-fold increase in the areal density of FF microrods [37].

Furthermore, the interaction between the double layers is very important for the device's performance. When the PENGs are based on FF microrods with the same polarization direction (head-to-head), the voltage from each layer contribute constructively to the device's voltage output. However, the device's current is in general less than the largest value of two layers. In comparison, when the PENGs is based on FF microrods with opposite polarization directions (head-to-tail), the current from each layer contribute constructively to the device's current output. However, the device's voltage is in general less than the largest value of two layers. The different behavior of the device's voltage output and current output is caused by the interaction of the double layers, and such interaction needs to be considered during device design.

The double-layer structured FF peptide microrods improve the performance of PENGs through the synchronous mechanical driving and constructive integration. However, the cost efficiency and scalability of PENGs is a challenge for long-term applications. We have also some thoughts on this. In the design of piezoelectric nanogenerators, the selection of a robust encapsulation with matching mechanical properties needs to be developed to improve the durability and stability in relatively harsh environments such as high temperatures. Additionally, structural measures should be taken to enhance the durability of piezoelectric nanogenerators. For example, optimizing the structural design of the devices can reduce material fatigue caused by prolonged repetitive stress. Moreover, adopting a modular design allows for the easy replacement of damaged parts without the need for a complete overhaul, thereby improving the devices' maintainability and reducing long-term operational costs.

## 3 Conclusion

In summary, we developed double-layer structured FF peptide microrod arrays with each layer's polarization independently controlled, and we demonstrated a strategy to improve the power output of PENGs through the synchronous mechanical driving and constructive integration. The double-layer structured FF peptide microrods are obtained through regulating temperature and humidity, and polarization of each layer is independently controlled by applying an appropriate electric field during the self-assembly process. Applying the same electric field during each layer's growth and connecting them in serial allow the double-layer structured PENG to produce an open-circuit voltage of 2.05 V, doubling the voltage output from a single-layer-based device. Similarly, the output current can also be doubled when opposite electric fields are applied during the growth and two layers are connected in parallel. This work paves the way to the growth of multi-layer peptide arrays and facilitates the design and integration of piezoelectric peptide-based devices.

## 4 Materials and methods

### 4.1 Fabrication of double-layer structured FF peptide microrod arrays

FF peptide microrod arrays were fabricated as previously reported [25]. Briefly, lyophilized FF powders (Absin) were dissolved in 1,1,1,3,3,3-hexafluoro-2-propanol (Aladdin) to form a solution at a concentration of 45 mg *L*^−1^. The amorphous film was prepared by dripping the 20 µL solution onto a 1.25 cm × 1.25 cm Ag-coated glass substrate in a desiccator. The seed film was obtained by crystallizing the amorphous film in a humid environment for 60 s. An FF water solution with a concentration of 1.75 mg mL^−1^ was obtained by dissolving lyophilized FF powders in water at 80 °C until it became clear. The FF water solution was placed in a Petri dish between two aluminum plates in a 55 °C oven. The FF seed film was suspended on the surface of the FF water solution. FF microrod arrays were obtained after three hours. An electric field was applied during the self-assembly of the FF peptides. Next, PDMS was spin-coated onto the first-layer structured FF microrod arrays at a speed of 3000 rpm, and the Ag electrode was evaporated on the PDMS layer. The second layer of FF peptide microrod arrays was obtained using the above-mentioned method.

### 4.2 Characterization of double-layer structured FF peptide microrod arrays

The micromorphology of the samples was measured using an optical microscope (LV-UEPI-N) and a scanning electron microscope (SEM, Apreo+HiVac). The structure of the samples was analyzed by X-ray diffraction (XRD, D8 Advance) using Cu Kα radiation in the angle range of 5° to 30°. The piezoelectric properties of the FF peptide microrod arrays were determined by atomic force microscopy (AFM, CypherES) equipped with a piezoresponse force module (PFM). Furthermore, the piezoelectric constant of d_33_ was tested at room temperature using a quasi–static piezoelectric d_33_–meter (Model ZJ–3A, Institute of Acoustics, Chinese Academy of Science, China).

### 4.3 Fabrication of double-layer structured PENGs

A substrate with the double-layer structured FF peptide microrods was attached to a 2.5 cm × 2.5 cm glass plate for easy manipulation. A PDMS film was spin-coated on the surface of the FF peptide microrod arrays as a protective layer. An Ag-coated glass substrate with a PDMS film was placed tightly over the FF peptide microrod arrays and secured with Kapton tape as the top electrode. Silver paste connected three wires to the top, middle, and bottom electrodes to form a double-layer structured PENG.

### 4.4 Characterization of double-layer structured PENGs

The double-layer structured PENG was mounted on a steel stage, and an external force was applied to the PENG by a linear motor (E100-RD-HC type with force control, LinMot). The output voltages and currents of the PENG were recorded with an electrometer (Keithley 6517B). The PENG was placed in a Faraday cage to avoid environmental disturbances.

## Declaration of competing interest

The authors declare that they have no conflicts of interest in this work.

## Appendix Supplementary materials

Image, application 1
