# Supplementary material for: Development of double-layer FF peptide microrod arrays for high performance piezoelectric nanogenerators
Source: Fundam Res. 2024 Aug 5;6(1):416–22. doi: 10.1016/j.fmre.2024.07.009 (PMC12869740; doi:10.1016/j.fmre.2024.07.009)
Supplement: Supplementary file 1 [file mmc1.docx]

Supporting Information

**Development of double-layer FF peptide microrod arrays for high performance piezoelectric nanogenerators**

Jiaojiao Zhang^1#^, Jing Liu^1#^, Wen Hu^1^, Xue Jiang^1^, Long Zhou^1^, Yumin Tang^2^, Zhong Lin Wang^1,3,4^*, Rusen Yang^1^*

^1^School of Advanced Materials and Nanotechnology, Xidian University, Xi’an 710071, China.

^2^Zhejiang Cachi New Energy Technology Co., Ltd., Huzhou 313100, China.

^3^Beijing Institute of Nanoenergy and Nanosystems, Chinese Academy of Sciences, Beijing 101400, China.

^4^School of Materials Science and Engineering, Georgia Institute of Technology, Atlanta, GA 30332-0245, United States.

*Corresponding author. E-mail: [rsyang@xidian.edu.cn](mailto:rsyang@xidian.edu.cn) (R. Yang), [zhong.wang@mse.gatech.edu](mailto:zhong.wang@mse.gatech.edu) (Z.L Wang)

**Fig. S1** Growth of vertical FF peptide microrod arrays with controlled polarization. The arrow pointing up represents a positive electric field growth, and the arrow pointing down represents a negative electric field growth.

**Fig. S2** Crystallization of the FF seed layer under different temperature and humidity conditions. (a-c) Optical images of the FF seed layer grown at 30 ºC with humidity of (a) ~80%. (b) ~90%. (c) ~100%. (d-f) Optical images of the FF seed layer grown at (d) 30 ºC. (e) 40 ºC. (f) 50 ºC.

**Fig. S3** (a) The FF microrod array after spin-coating with PDMS. (b) Deposition of the intermediate electrode layer.

**Fig. S4** Optical images of the second FF seed layer at 30 ºC with humidity of ~100%.


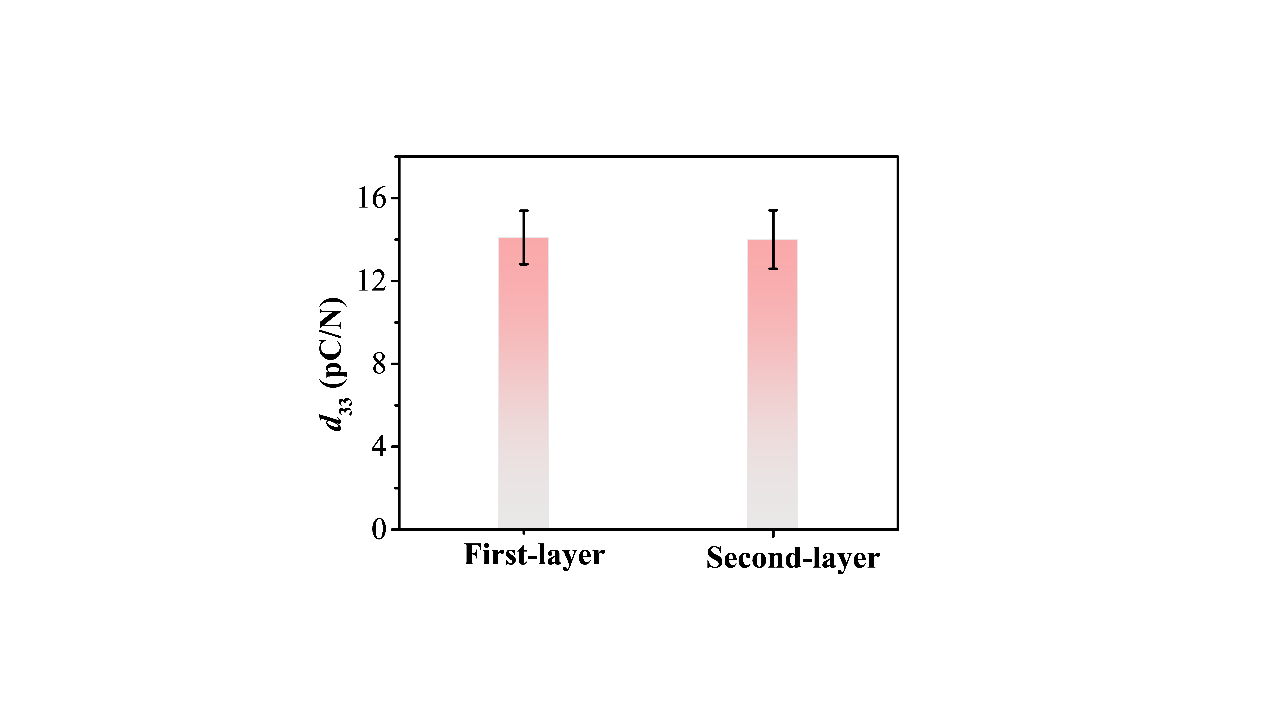


**Fig. S5** The d_33_ of FF microrods from the first layer and from the second layer.

**Fig. S6** The output performance of PENGs based on the double-layer structured FF peptide microrods of the first layer grown with positive electric field and the second layer with negative electric field. (a) Output voltages and (b) currents of first-layer, second-layer, and double-layer structured PENGs.

**Fig. S7** The output voltages of PENG devices based on FF peptide microrods of the first layer grown with the positive electric field and the second layer with the negative electric field in reverse connection.

**Fig. S8** Dependence of open-circuit voltages and short-circuit currents on the applied force of double-layer structured PENGs based on FF peptide microrods from (a) the first-layer grown with a positive electric field, (b) the second-layer grown with a negative electric field, and (c) the double-layer structured PENGs.


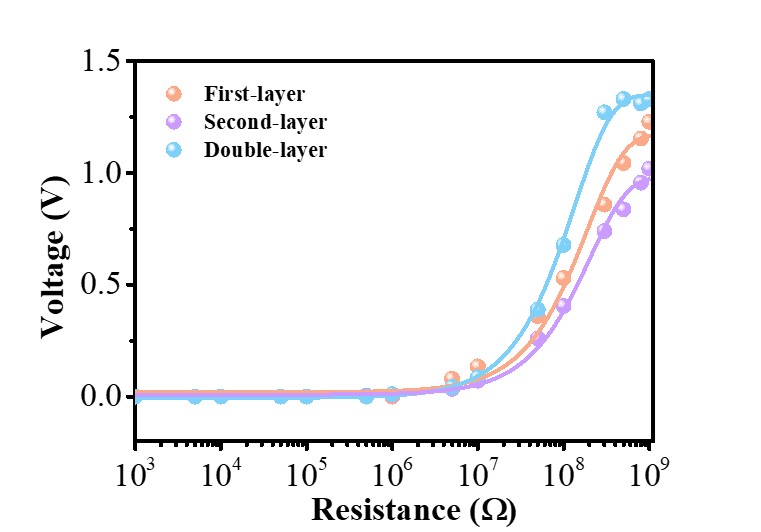


**Fig. S9** The voltage of PENG with different load resistances based on the double-layer structured FF peptide microrod arrays of the first layer grown with positive electric field and the second layer with negative electric field.


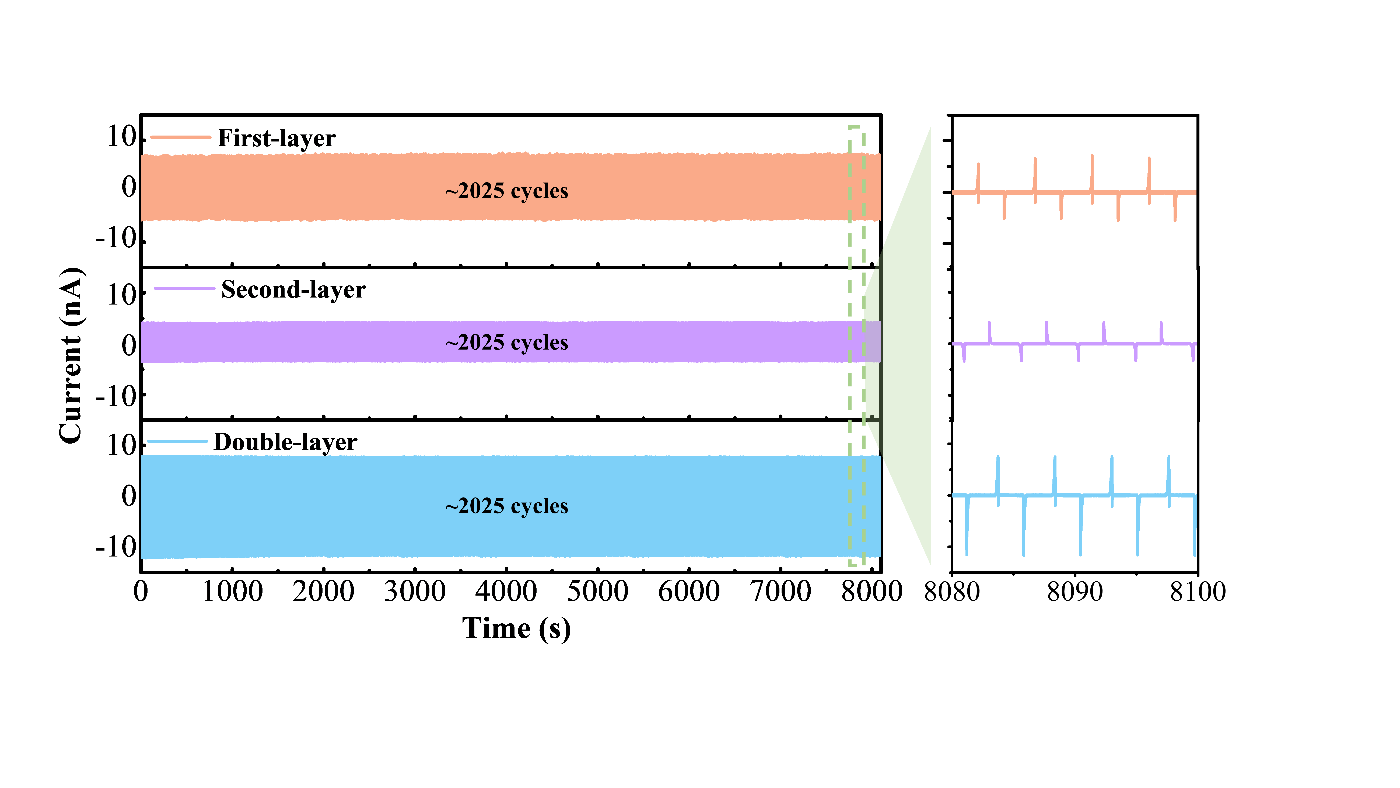


**Fig. S10** Current outputs of the double-layer structured PENG based on FF peptide microrods of the first layer grown with a positive electric field and the second layer with a negative electric field for about 2025 cycles.

**Fig. S11** Electrical outputs of the double-layer structured PENG based on FF peptide microrods with positive electric field applied to each layer growth. (a) Output voltages and (b) currents of the first-layer, second-layer, and double-layer structured PENG devices.

**Fig. S12** Output voltages obtained from PENG devices based on FF peptide microrods with positive electric field applied to each layer growth in reverse connection.

**Fig. S13** Dependence of open-circuit voltages and short-circuit currents on the applied force of double-layer structured PENGs based on FF peptide microrods from (a) the first-layer grown with a positive electric field, (b) the second-layer grown with a positive electric field, and (c) the double-layer structured PENGs.


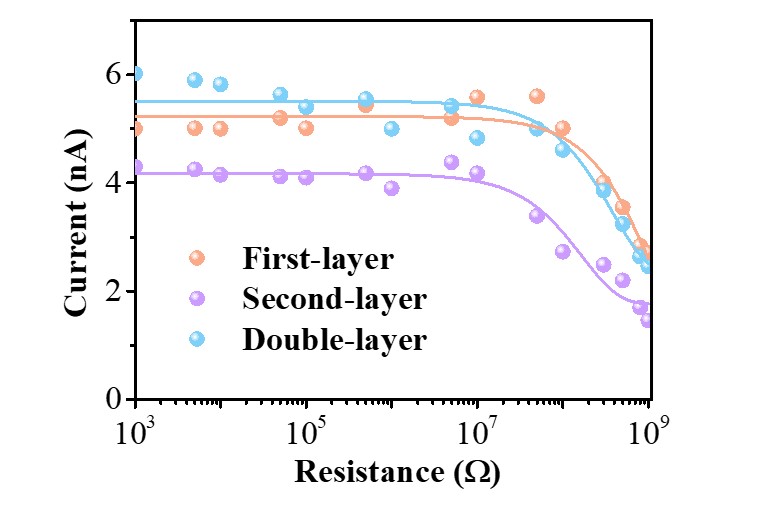


**Fig. S14** The current of PENG with different load resistances based on the double-layer structured FF peptide microrod arrays with positive electric field applied to each layer growth.
